# Short-Term Rationing of Combination Antiretroviral Therapy: Impact on Morbidity, Mortality, and Loss to Follow-Up in a Large HIV Treatment Program in Western Kenya

**DOI:** 10.1155/2012/814564

**Published:** 2012-05-29

**Authors:** April J. Bell, Kara Wools-Kaloustian, Sylvester Kimaiyo, Hai Liu, Adrian Katschke, Changyu Shen, Gilbert Simiyu, Beverly S. Musick, John E. Sidle, Abraham Siika, Paula Braitstein

**Affiliations:** ^1^Indiana University School of Medicine, 1001 West 10th Street, OPW M200, Indianapolis, IN 46202, USA; ^2^USAID-Academic Model for Providing Access to Healthcare (AMPATH), P.O. Box 5760-30100, Eldoret, Kenya; ^3^Moi University School of Medicine, P.O. Box 4606-30100, Eldoret, Kenya; ^4^Regenstrief Institute Incorporated, 1050 Wishard Boulevard, RG 5, Indianapolis, IN 46202, USA; ^5^Dalla Lana School of Public Health, University of Toronto, 6th Floor, Health Sciences Building, 155 College Street, Toronto, ON, Canada M5T 3M7

## Abstract

*Background*. There was a 6-month shortage of antiretrovirals (cART) in Kenya. *Methods*. We assessed morbidity, mortality, and loss to follow-up (LTFU) in this retrospective analysis of adults who were enrolled during the six-month period with restricted cART (cap) or the six months prior (pre-cap) and eligible for cART at enrollment by the pre-cap standard. Cox models were used to adjust for potential confounders. *Results*. 9009 adults were eligible for analysis: 4,714 pre-cap and 4,295 during the cap. Median number of days from enrollment to cART initiation was 42 pre-cap and 56 for the cap (*P* < 0.001). After adjustment, individuals in the cap were at higher risk of mortality (HR = 1.21; 95% CI : 1.06–1.39) and LTFU (HR = 1.12; 95% CI : 1.04–1.22). There was no difference between the groups in their risk of developing a new AIDS-defining illness (HR = 0.92 95% CI 0.82–1.03). *Conclusions*. Rationing of cART, even for a relatively short period of six months, led to clinically adverse outcomes.

## 1. Introduction

Since the beginning of the HIV pandemic, almost 60 million people have been infected with HIV and 25 million have died from HIV-associated illnesses [1]. Sub-Saharan Africa is the region most affected by the pandemic and is home to 68% of all people living with HIV worldwide [1]. Since 2002, the international drive to scale up antiretroviral treatment has gained tremendous momentum [2], and by the close of 2009, an estimated 5.2 million persons were receiving combination antiretroviral treatment (cART). While this represents important progress, this still is only about 35% of the people who are estimated in need of treatment according to current standards of care [1].

HIV/AIDS has largely been transformed into a manageable, chronic disease for those with access to cART [3]. The clinical benefits of cART for individuals living in resource-poor settings, including slow disease progression and reduced mortality, have been documented in multiple studies [4–6]. More specifically, recent data from Uganda demonstrated that morbidity in HIV-infected individuals decreased after the introduction of antiretroviral therapy (ART); the decline became more apparent with increasing duration on ART [7]. Nonetheless, questions remain about the optimal time to begin treatment [8, 9].

Recent observational data from North America show that the risk of death increased by 69% when initiation of therapy is delayed until after the CD4 count drops below 350 cells/*μ*L [8]. The nadir CD4 count is predictive of the benefit that will be gained from cART initiation [8, 10]. Prior to November 2009, guidelines for low-income settings recommended initiating treatment when a patient's CD4 count dropped below 200 cells/*μ*L [11]. The guidelines were revised in November 2009 and now recommend initiation of treatment earlier in the course of the disease, when the CD4 cell count falls below 350 cells/*μ*L [12]. Unfortunately, many clinics and treatment providers are already overwhelmed with the number patients presenting for treatment [13]. The capacity of the infrastructure to manage HIV as a chronic illness is stretched, and though the guidelines for cART initiation have been expanded, the capacity of clinics in resource-constrained settings to meet the increased demand is not clear [14]. Furthermore, international commitments to continued funding and expansion of HIV treatment programs are waning in the face of global financial constraints [15, 16]. Rationing and waitlisting for treatment initiation may continue to be a reality for many antiretroviral treatment programs given these circumstances. The impact of rationing treatment on patient clinical outcomes is not well described.

From March 12, 2007 through August 31, 2007, Kenya experienced a shortage of antiretroviral medications. The United States Agency for International Development (USAID) funded Academic Model Providing Access to Healthcare (AMPATH) was asked by the Government of Kenya to limit new cART initiations. The program agreed to limit new cART starts to patients with CD4 < 100 cells/*μ*L, effectively “capping” new cART initiations for a period of 6 months. The primary objective of this analysis was to evaluate the impact of this restriction on morbidity, mortality, and loss to follow-up among HIV-infected patients who were eligible for cART. Our secondary objective was to determine factors associated with actually receiving cART among this population.

## 2. Methods

### 2.1. Study Design

 This retrospective observational study was approved by the Indiana University School of Medicine Institutional Review Board and the Moi University Institutional Research and Ethics Committee. Informed consent was waived as a part of a general approval for conducting retrospective analyses with de-identified data collected as a part of routine care.

### 2.2. Study Setting

 AMPATH, headquartered in Eldoret, Kenya, was established in 2001 as a partnership between Indiana University School of Medicine, Moi University School of Medicine, and the Moi Teaching and Referral Hospital. In 2004, AMPATH received funding from the United States Presidential Emergency Plan for AIDS Relief (PEPFAR) to create the USAID-AMPATH Partnership. The original goal of the program was to establish an HIV care network to serve the needs of patients in western Kenya. To date, the program has enrolled more than 135,000 patients in 25 Ministry of Health facilities and numerous satellite clinics around western Kenya. All HIV and tuberculosis-related care and treatment are provided without cost to patients through USAID-AMPATH and the Kenyan Department of Leprosy, TB, and Lung Disease.

### 2.3. Study Population

 The study population was limited to patients who were not pregnant, aged 14 or older at enrollment, and who were eligible for cART at enrollment according to the pre-cap standard. Specifically, patients were eligible for cART if they had (1) CD4 < 200 cells/*μ*L or (2) WHO stage IV illness or (3) WHO stage III AND CD4 < 350 cells/*μ*L. These criteria are consistent with the 2006 HIV treatment guidelines from the World Health Organization [17]. Pregnant women were excluded from the analysis because they were exempted from the cap criteria (i.e., they continued to be initiated on cART as usual). There were two populations of eligible patients for this analysis: those who enrolled during the six months prior to the restricted cART period (September 1, 2006–March 11, 2007, called the “pre-cap” period) and those who enrolled during the restricted cART period (March 12–August 31, 2007, called the “cap” period).

### 2.4. Data Collection and Management

 Clinicians completed standardized forms capturing demographic, clinical, and pharmacologic information at each patient visit. These data are then hand-entered into the AMPATH Medical Record System (AMRS), a secure computerized database designed for clinical management, with data entry validated by random review of 10% of the forms entered [18, 19]. At the time of registration, patients are provided with a unique identifying number. For this study, all data were de-identified before analysis.

### 2.5. Outcomes and Explanatory Variables

 The primary outcomes for this analysis were morbidity, mortality (from all causes) and loss to follow-up (LTFU). Morbidity is defined as a new WHO Stage III or IV illness 60 days after enrollment to ensure that disease prevalent at enrolment was not accidentally counted as an incident infection due to delayed provider ascertainment. LTFU is defined as being absent from clinic for at least three months if on cART at the last visit and with no indication that the patient had died, or if not on cART at last visit, absent from clinic for at least six months, with no indication that the patient had died. AMPATH has a Standard Operating Procedure for reporting deaths, which includes a standardized death reporting form that is used for documentation of deaths by all clinic personnel. AMPATH also has an active peer-led outreach program which assists with death ascertainment. Outreach workers complete locator information for all new and returning patients in the clinical care program. The locator card includes contact information and a map to the patient's residence and is used to find the patient in the event of a missed appointment. The AMPATH Medical Record System (AMRS) produces a daily list of patients scheduled for appointments and patients that miss their appointment are listed for outreach based on a three-tier triage algorithm. Adult patients on CART for less than three months are given priority. Outreach efforts for these patients are to commence within 24 hours of a missed appointment with a goal of locating the patient within seven days. For patients receiving CART for over three months, outreach is activated within seven days after a missed appointment. Individuals who do not receive CART are given 28 days from the missed appointment prior to initiation of outreach activities. However, there is undoubtedly underascertainment of deaths among patients LTFU—a recent evaluation of patients LTFU found that 20% of patients LTFU were in fact deceased.

Independent variables were both sociodemographic and clinical. We hypothesized that the following variables were actual or potential confounders of the relationship between the cap and death or LTFU and so were included in the analyses: age (continuous), sex (male/female), WHO stage (I, II, III, IV) and CD4 count (continuous) at enrollment, use of Cotrimoxazole or Dapsone at enrollment, whether the patient received TB treatment at enrolment, whether the patient was attending an urban or a rural clinic, and the time required for the patient to travel to clinic (<30 minutes, 30–60 minutes, 1-2 hours, >2 hours).

### 2.6. Statistical Methods

Enrollment cohort characteristics of the “pre-cap” group and “cap” group were compared using Fisher's Exact test for categorical variables and Kruskal-Wallis test for continuous variables. The Kaplan-Meier method was used to estimate the survival functions of time to morbidity, loss to follow-up and mortality. Time zero was the date of enrollment for the loss to follow-up and mortality analyses and 60 days after enrollment in the morbidity model. The event date of loss to follow-up was the date of the last clinic visit recorded in the database. Data on individuals known to have died within 3 months of their last clinic visit (if the patients were on cART, or 6 months if not on cART) were censored at the date of their last visit in the LTFU model. The patients on cART who are known to have died more than 3 months after their last clinic visit (or more than 6 months if not on cART) were treated as LTFU at the date of their last visit. Data for those who were still alive and not lost to follow-up by the administrative closure of the database were censored at the date of the last clinic visit. Survival distributions were compared using the Wilcoxon Log Rank test.

Cox Proportional Hazard models were used to calculate adjusted hazard ratios with 95% confidence intervals in individual predictive models for the primary outcomes (adjusting for the covariates described previously). In secondary analysis we explored the factors associated with cART initiation using Cox models. Statistical significance in the Cox model was assessed by the Wald test. Variables, if statistically significant at an alpha of 0.05 in the univariate model or, if believed to be potential confounders, were entered into the final model. All covariates in the tables were adjusted for in the multivariate models.

All statistical analyses were performed using R software (version 2.13.1; Vienna, Austria) and SAS (version 9.2; SAS Institute, Cary, NC).

## 3. Results

### 3.1. Participants

There were 9,009 adults eligible for analysis. Among whom, 60.2% were women, with a median age of 37.0 (interquartile range, IQR 30.9, 44.1). The median CD4 count at enrollment was 96 (IQR: 40, 162) cells/*μ*L. Of the 9,009, 4,714 individuals were enrolled in the pre-cap period and 4,295 were enrolled during the cap period. Patients were followed for a median of 559 days (700 days in the pre-cap group and 512 for the cap group). This difference is to be expected since the pre-cap group were enrolled in the 6 months prior to the start of the cap period and thus had a longer potential follow-up period.

The clinical and sociodemographic characteristics of both groups are summarized in Table 1. Median CD4 count at enrolment was similar between the two groups (99 versus 94). The groups were well balanced on all enrollment characteristics except for use of Cotrimoxazole or Dapsone prophylaxis (81.7% in the pre-cap group versus 76.4% in the cap group, *P* < 0.001).

### 3.2. Time to cART Initiation

There were 3,684 (78.2%) individuals who initiated cART in the pre-cap group and 3,126 (72.8%) in the cap cohort (*P* < 0.001). The median number of days from enrollment to cART initiation was 50 days for the pre-cap cohort and 70 days for the cap cohort (*P* < 0.001). Factors associated with receiving cART are summarized in Table 2. Individuals were more likely to receive cART if they were receiving Cotrimoxazole or Dapsone (*P* < 0.001), if they were male (*P* < 0.001), if they were WHO Stage II, III, or IV (compared to stage I), or if they attended an urban clinic (*P* = 0.030). Patients were less likely to receive cART if they were enrolled during the cap period (*P* < 0.001), and if they were receiving TB treatment (*P* < 0.001). For each one cell increase in CD4 count there was a 0.5% reduction in the likelihood of getting cART (Hazard Ratio, HR: 0.995, 95% confidence interval, CI: 0.995-0.996).

### 3.3. Morbidity

**Figure 1:**
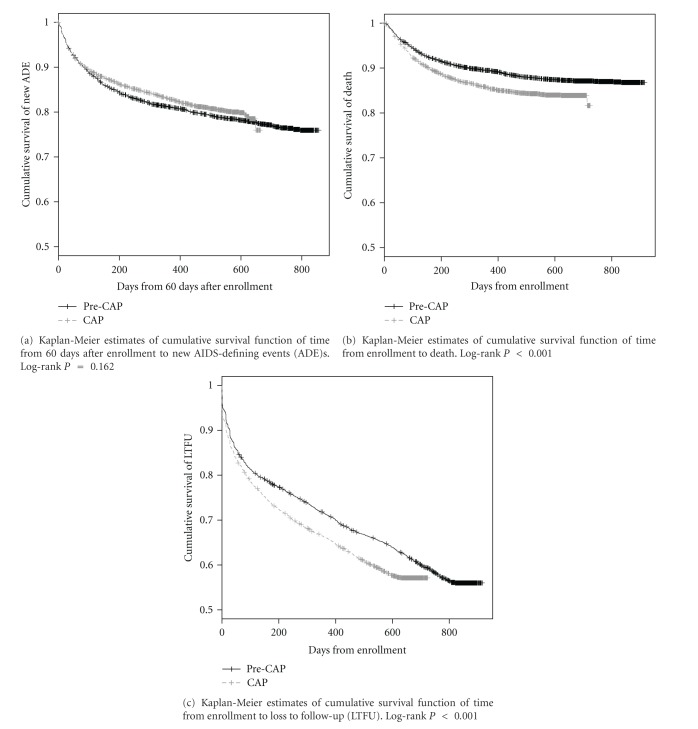
Kaplan-Meier estimates of cumulative survival function of time to (a) new AIDS defining event (ADE); (b) death; (c) loss to follow-up.

There were 1,358 new AIDS defining events during the follow-up period including 573 among the cap group and 785 among the pre-cap group. After adjustment for potential confounders, there was no effect of the cap on the risk of developing a new WHO Stage III or IV illness (HR: 0.92; 95% CI: 0.82, 1.03) (Figure 1(a), Table 3).

### 3.4. Mortality

There were 1030 deaths during the follow-up period, including 538 among the cap group and 492 among the pre-cap group. Among patients who died, the median number of days to death in the cap group was 94 (IQR: 46, 201) compared to the pre-cap group which was 111 (44, 244). Among the 492 deaths in the pre-cap group, 191 (38.8%) died before starting cART, while for the cap group, 261 out of 538 deaths (48.5%) were before cART initiation (*P* = 0.002). The 1-year survival rate in the pre-cap group was 89.5% (95% CI: 88.5%, 90.4%), compared to 85.6% (84.4%, 86.8%) in the cap group (Figure 1(b)). After adjusting for covariates, the cap group had a significantly higher risk of mortality (HR = 1.21; 95% CI: 1.06, 1.39) compared to the pre-cap group (Table 3).

### 3.5. Loss to Follow-Up

There were 3,537 patients lost to follow-up, including 1,665 in the cap group and 1,872 in the pre-cap group. The adjusted relative risk of becoming loss to follow-up was 1.12 (1.04, 1.22) times greater for those patients enrolled during the cap period versus the pre-cap period (Figure 1(c), Table 3).

### 3.6. Sub-Analysis for Pneumocystis Prophylaxis

The patients in the cap group were somewhat less likely to receive Cotrimoxazole or Dapsone compared to the pre-cap group. To explore whether residual confounding arising from this explains the increases in mortality and loss to follow-up among the cap group, a sub-analysis was conducted in which we restricted the population to only those who received Cotrimoxazole or Dapsone. Our findings were marginally affected as a result (Mortality HR: 1.20, 95% CI: 1.04–1.40; LTFU HR: 1.18, 95% CI: 1.08–1.28).

## 4. Discussion

These data suggest that even a relatively brief restriction of cART initiation among otherwise eligible patients independently contributes to a higher risk of mortality and loss to follow-up. This is in spite of triaging the sicker patients (as measured by CD4 and WHO clinical stage) to receive cART before the healthier ones. These findings underscore the negative effects that can be expected from even a short-term delay in the initiation of cART among otherwise eligible patients. We believe that our study did not show an effect of increased morbidity while still showing increased mortality and LTFU because patients who developed new AIDS-defining illnesses died or became lost to follow-up before these illnesses could be diagnosed and/or documented in the clinical encounter.

 In addition to the poor clinical outcomes arising directly from the delays, it can be expected that the long-term clinical effectiveness of cART once initiated may also be adversely affected by virtue of patients initiating treatment at more advanced levels of immune suppression [20]. Furthermore, there are important implications of delayed cART initiation on transmission of HIV and tuberculosis [3]. Prevention of mother-to-child transmission [21], postexposure prophylaxis, and most recently HPTN 052 [22] have demonstrated the dramatic impact that combination antiretroviral treatment can have on HIV transmission. As such delayed cART initiation has multiple downstream consequences both for the patient themselves and for their partners, offspring and community. Given these recent data on treatment for prevention as well as data on the clinical consequence of ART delay, it is imperative that global community continues its commitment to providing cART in resource constrained settings. These data illustrate the stark consequences of failing to live up to these commitments and will put clinicians on the front-lines back in the unenviable position endured prior to the global scale-up of antiretroviral treatment delivery: having to choose who will live, and who will not.

There are several strengths to this study. The first is that the USAID-AMPATH Partnership is a large clinical population, covering much of western Kenya, in both urban and rural settings. As a result, this sample provided ample statistical power to answer our primary question and is broadly generalizable to other sub-Saharan Africa settings. Second, AMPATH services are free to patients, eliminating confounding due to fee for service care structures. Last, because there were no pharmacy stock-outs during the study period (other than the one that caused the circumstances for the comparison), our findings are not confounded by other disruptions in the supply chain.

Limitations to this analysis include the potential random misclassification due to clinician error in recording. Similarly, incomplete ascertainment of outcomes may have particularly affected the morbidity analysis. Third, this is a retrospective study, with its inherent limitations including reliance on accuracy of written record and incomplete data. Fourth, there may be prescription bias, in which providers started cART on patients who did not meet the initiation guidelines but whom they felt would benefit from treatment nevertheless. If this occurred, it will have biased the results towards the null.

## 5. Conclusions

Given the certainty that more people will become eligible as early start strategies are implemented and that early start, itself, is a prevention strategy, this study highlights the need for international funding organizations and national governments to continue and expand their commitment to HIV care and treatment. Treatment programs can make the best use of resources through task-shifting [23–25] and other innovative models, such as community ART group models [26], to ensure the continuous provision of cART to those in need in resource-limited settings. The stakes for patients are high and these data demonstrate that even small delays in treatment initiation among patients who are immune suppressed can be fatal. As the HIV pandemic begins to stabilize in sub-Saharan Africa, and as the population and economic benefits of treating HIV infection begin to accrue there as elsewhere [20, 27], these data remind us that turning back the clock and restricting even sick patients from accessing treatment is simply not a reasonable option.

## Figures and Tables

**Table 1:** Enrollment sociodemographics and clinic characteristics of the comparison groups.

Variable	Pre-cap group (*n* = 4714), *n* (%)	Cap group (*n* = 4295), *n* (%)	*P *value
Sex			
Male	1849 (39.2)	1741 (40.5)	0.212
Female	2865 (60.8)	2554 (59.5)	
Missing	0 (0)	0 (0)	

Age^a^			
Median	37.0	36.9	0.255
IQR	31.0–44.1	30.6–44.0	
Missing	0 (0)	0 (0)	

Clinic location^a^			
Urban	2315 (49.1)	2176 (50.7)	0.146
Rural	2399 (50.9)	2119 (49.3)	
Missing	0 (0)	0 (0)	

Travel time to clinic^a^			
<30 minutes	1093 (23.4)	962 (23.1)	0.100
30–60 minutes	1467 (31.5)	1400 (33.6)	
1-2 hours	1266 (27.2)	1127 (27.0)	
>2 hours	836 (17.9)	683 (16.4)	
Missing	52 (1.1)	123 (2.9)	

WHO clinical stage^a^			
I	637 (15.1)	533 (14.5)	0.780
II	839 (19.8)	754 (20.6)	
III	2278 (53.9)	1977 (54.0)	
IV	476 (11.3)	400 (10.9)	
Missing	484 (10.3)	631 (14.7)	

CD4^a^			
Median	99.0	94.0	0.081
IQR	41.0–163	38.0–161	
Missing	59 (1.3)	49 (1.1)	

Use of Cotrimoxazole or Dapsone			
Yes	3851 (81.7)	3282 (76.4)	<0.001
No	863 (18.3)	1013 (23.6)	
Missing	0 (0)	0 (0)	

TB treatment^a^			
Yes	1180 (25.0)	1121 (26.1)	0.246
No	3534 (75.0)	3174 (73.9)	
Missing	0 (0)	0 (0)	

^a^At Enrollment.

**Table 2:** Cox proportional (unadjusted and adjusted) hazard ratios for predictors of cart initiation.

Covariate	Univariate analysis			Multivariate analysis^b^		
	Unadjusted hazard ratio	95% confidence interval (CI)	*P* value	Adjusted hazard ratio (AHR)	95% Confidence interval (CI)	*P* value
Enrolled in the cap period	0.86	0.82, 0.90	<0.001	0.87	0.83, 0.92	<0.001
Use of Cotrimoxazole or Dapsone^a^	3.22	2.98, 3.48	<0.001	2.08	1.89, 2.29	<0.001
On TB treatment^a^	0.77	0.73, 0.82	<0.001	0.60	0.56, 0.64	<0.001
Male gender	1.13	1.08, 1.19	<0.001	1.14	1.09, 1.21	<0.001
Age (per year increase)^a^	1.00	1.00, 1.00	0.100	1.00	1.00, 1.00	0.950
CD4 (per cell increase)^a^	0.995	0.994, 0.995	<0.001	0.995	0.995, 0.996	<0.001
WHO Stage I^a^	1.00			1.00		
WHO Stage II^a^	1.16	1.07, 1.26	<0.001	1.08	1.00, 1.17	0.053
WHO Stage III^a^	0.89	0.83, 0.95	0.001	1.20	1.11, 1.29	<0.001
WHO Stage IV^a^	1.33	1.20, 1.46	<0.001	1.86	1.67, 2.07	<0.001
Urban Clinic^a^	1.03	0.99, 1.09	0.162	1.06	1.01, 1.11	0.030
Travel time < 30 min	1.00			1.00		
Travel time 30–60 min	1.02	0.96, 1.09	0.494	0.99	0.93, 1.06	0.749
Travel time 1-2 hr	1.05	0.98, 1.12	0.151	0.99	0.92, 1.06	0.742
Travel time > 2 hr	1.11	1.03, 1.20	0.008	1.05	0.97, 1.14	0.246

^a^At Enrollment.

^b^Adjusted for all covariates.

**Table 3:** Cox proportional (unadjusted and adjusted) hazard ratios and 95% confidence intervals for morbidity, loss to follow-up, and mortality.

	Morbidity				Loss to follow-up				Mortality			
	Unadjusted		Adjusted^b^		Unadjusted		Adjusted^b^		Unadjusted		Adjusted^b^	

Covariate	HR (95% CI)	*P *value	HR (95% CI)	*P *value	HR (95% CI)	*P *value	HR (95% CI)	*P *value	HR (95% CI)	*P *value	HR (95% CI)	*P *value
Enrolled during cap period	0.93 (0.83, 1.03)	0.162	0.92 (0.82, 1.03)	0.152	1.19 (1.11, 1.27)	<0.001	1.12 (1.04, 1.22)	0.004	1.31 (1.16, 1.48)	<0.001	1.21 (1.06, 1.39)	0.006
Use of Cotrimoxazole or Dapsone^a^	0.66 (0.58, 0.76)	<0.001	0.67 (0.55, 0.80)	<0.001	0.39 (0.36, 0.42)	<0.001	0.58 (0.52, 0.65)	<0.001	0.49 (0.42, 0.56)	<0.001	0.46 (0.37, 0.57)	<0.001
TB treatment^a^	0.82 (0.72, 0.93)	0.003	0.68 (0.59, 0.78)	<0.001	1.04 (0.97, 1.12)	0.299	0.99 (0.90, 1.09)	0.850	1.11 (0.97, 1.27)	0.136	0.84 (0.72, 0.99)	0.033
Male gender	0.98 (0.88, 1.09)	0.718	0.95 (0.85, 1.07)	0.418	1.05 (0.98, 1.12)	0.187	1.01 (0.93, 1.10)	0.760	1.48 (1.31, 1.67)	<0.001	1.36 (1.19, 1.57)	<0.001
Age (per year increase)^a^	1.00 (0.99, 1.00)	0.333	1.00 (0.99, 1.01)	0.772	0.99 (0.98, 0.99)	<0.001	0.99 (0.98, 0.99)	<0.001	1.00 (1.00, 1.01)	0.388	1.00 (1.00, 1.01)	0.484
CD4 (per cell increase)^a^	0.999 (0.999, 1.000)	0.006	0.998 (0.998, 0.999)	<0.001	0.999 (0.999, 0.999)	<0.001	0.999 (0.999, 0.999)	<0.001	0.995 (0.994, 0.996)	<0.001	0.995 (0.994, 0.996)	<0.001
WHO Stage I^a^	1.00		1.00		1.00		1.00		1.00		1.00	
WHO Stage II^a^	1.24 (1.01, 1.51)	0.038	1.23 (1.01, 1.51)	0.044	0.95 (0.83, 1.09)	0.454	0.96 (0.83, 1.10)	0.543	1.19 (0.87, 1.63)	0.279	1.10 (0.80, 1.50)	0.572
WHO Stage III^a^	1.49 (1.26, 1.78)	<0.001	1.64 (1.36, 1.96)	<0.001	1.32 (1.18, 1.49)	<0.001	1.24 (1.10, 1.40)	<0.001	2.62 (2.02, 3.40)	<0.001	2.44 (1.87, 3.19)	<0.001
WHO Stage IV^a^	2.10 (1.68, 2.62)	<0.001	2.55 (2.01, 3.24)	<0.001	2.08 (1.80, 2.40)	<0.001	1.98 (1.70, 2.31)	<0.001	4.51 (3.37, 6.04)	<0.001	4.41 (3.24, 5.99)	<0.001
Urban Clinic^a^	0.99 (0.89, 1.10)	0.857	0.99 (0.88, 1.11)	0.856	1.05 (0.99, 1.12)	0.128	1.02 (0.94, 1.10)	0.651	0.76 (0.68, 0.87)	<0.001	0.80 (0.70, 0.92)	0.002
Travel time < 30 min	1.00		1.00		1.00		1.00		1.00		1.00	
Travel time 30–60 min	0.93 (0.80, 1.07)	0.305	0.95 (0.81, 1.10)	0.491	1.13 (1.03, 1.24)	0.009	1.14 (1.03, 1.27)	0.014	1.36 (1.14, 1.62)	<0.001	1.18 (0.97, 1.43)	0.098
Travel time 1-2 hr	1.04 (0.90, 1.21)	0.598	1.01 (0.86, 1.18)	0.908	1.07 (0.97, 1.17)	0.204	1.07 (0.96, 1.20)	0.231	1.30 (1.08, 1.56)	0.005	1.09 (0.89, 1.34)	0.390
Travel time > 2 hr	0.98 (0.83, 1.17)	0.846	0.93 (0.78, 1.12)	0.464	1.34 (1.20, 1.48)	<0.001	1.40 (1.24, 1.58)	<0.001	1.38 (1.12, 1.68)	0.002	1.16 (0.93, 1.45)	0.192

^a^At enrollment.

^b^Adjusted for all covariates.
